# The Influence of the Drying Process on the Dissolution Time of Concentrated Chinese Medicine Pills: Roles of Textural Properties and Water Migration

**DOI:** 10.3390/pharmaceutics18050563

**Published:** 2026-04-30

**Authors:** Xiaojun Wang, Qinmin Meng, Xiaojian Luo, Yao Zhang, Jing Yang, Xiaoyong Rao, Yingming Zhang, Haowei Lu, Yan He, Wei Liu

**Affiliations:** 1 National Pharmaceutical Engineering Center for Solid Preparation in Chinese Herbal Medicine, Jiangxi University of Chinese Medicine, Nanchang 330006, China; wangxiaojun@jxutcm.edu.cn (X.W.); 19840211@jxutcm.edu.cn (X.L.); zhangyao@jxutcm.edu.cn (Y.Z.); yangjing1@jxutcm.edu.cn (J.Y.); 20141008@jxutcm.edu.cn (X.R.); 2 Beijing Yanjing Found Biological Technology Co., Ltd., Beijing 101300, China; zhongfahr@yanjing.com.cn; 3 Lanzhou Foci Pharmaceutical Co., Ltd., Lanzhou 730030, China; zhangyingming@fczy.cn; 4 Department of Pharmacy, The Affiliated Hospital of Jiangxi University of Chinese Medicine, Nanchang 330006, China; luhaowei@jxutcm.edu.cn

**Keywords:** Liuwei Dihuang concentrated pill, drying method, dissolution time, texture properties, water migration

## Abstract

**Objectives**: Concentrated pills, as a modernization and upgrade of traditional pills, have achieved significant advancements in dosage form. However, their extended disintegration and dispersion times have become a major limitation to their therapeutic efficacy. Therefore, an in-depth study and explanation of the dissolution mechanism of concentrated pills, along with the development of processing technology to control dissolution time, has emerged as a critical bottleneck in improving the quality of concentrated pills. **Methods**: In this study, the Liuwei Dihuang (LWDH) concentrated pill, derived from the classical Liuwei Dihuang pill, was selected as a representative model. Two drying methods—hot-air drying and hot air–microwave combined drying—were comparatively investigated to evaluate their effects on dissolution time. The dissolution behavior was elucidated by analyzing water migration during the dissolution process, as well as the textural properties and internal structural characteristics of the pills using Low-Field Nuclear Magnetic Resonance (LF-NMR), Micro-Computed Tomography (Micro-CT), texture analysis, and other modern techniques. **Results**: The results indicated that: (i) The rate of water absorption during the dissolution process of the LWDH pill was influenced by the number and size of the internal pores. (ii) Hot air–microwave combined drying results in more pores and faster dissolution. (iii) High-Performance Liquid Chromatography (HPLC) fingerprints showed no significant differences in the active ingredients between the samples. **Conclusions**: The drying method significantly affected the internal structure of the pills, suggesting that controlling the drying process could address the prolonged dissolution time of concentrated pills.

## 1. Introduction

Concentrated pills, a form of traditional Chinese medicine, were first recorded in Ge Hong’s Handbook of Prescriptions for Emergency during the Jin Dynasty. These pills are made by extracting and concentrating the ingredients from tablets or parts of tablets, then combining them with suitable excipients or fine tablet powders. The pills are bound using water, condensed honey, or a combination of honey and water. In modern times, while retaining the traditional advantages of these pills, the preparation process has been further innovated, and their scope of application expanded. This evolution has preserved the essence of traditional pills while integrating modern science and technology, significantly enhancing their therapeutic potential and application value.

Despite the convenience and popularity of concentrated pills, which benefit from their small size and ease of consumption, issues with unstable quality frequently arise due to factors such as production conditions and preparation processes. A particularly prominent issue is the substandard dissolution and dispersal time of the pills. However, dissolution is a critical step in the release and absorption of the active ingredients, which greatly influences the bioavailability and efficacy of the pills. Dissolution time is also a key indicator in the evaluation of concentrated pills. Therefore, optimizing the dissolution process is crucial to ensure the quality and efficacy of these pills [1]. Elucidating the dissolution mechanism and developing strategies to increase the dissolution rate could be key to resolving this issue.

**Figure 1:**
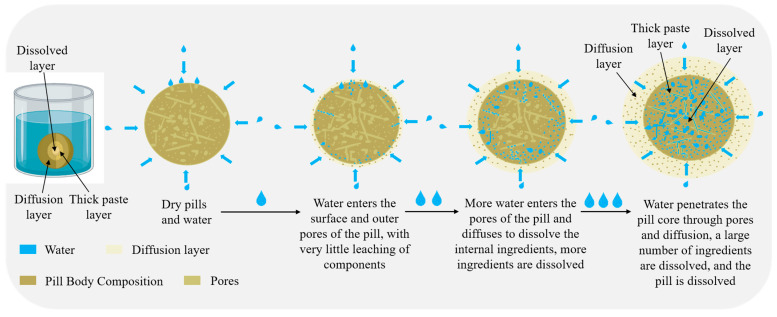
Schematic diagram of pill dissolution process.

At present, the mechanisms governing the dissolution and control of concentrated pills are poorly understood, and standard approaches provide limited basic information regarding the dissolution process [2], making it difficult to model drug dissolution effectively in vivo. The disintegration mechanism of tablets, dominated by the effects of soluble pores, solid bridge fracture, water absorption and swelling, and hygrothermal interactions, may be relevant to understanding the dissolution mechanism of concentrated pills [3,4]. However, the breakdown and release of active components from concentrated pills is more complex than the disintegration of chemical formulations. An in-depth study of the dissolution time of concentrated pills, including the interactions of wettability, capillarity, swelling, and dissolution on the pill’s surface, as well as an examination of the nature of the extracts, fine tablet powders, molding processes, and drying methods, could further elucidate the dissolution mechanism of concentrated pills [5] (Figure 1).

Excipient addition during formulation preparation and process optimization are two significant strategies for controlling dissolution capability [6,7,8]. However, increasing the number of excipients alters the specifications of the concentrated pills and increases the dosage required for patients. Furthermore, unlike tablets that undergo strong compression molding, the molding process of concentrated pills involves less pressure, and the addition of excipients may disrupt their structure, reducing formability and stability. Consequently, the use of disintegrants is typically avoided or minimized during production. Many studies are currently focused on the drying process of Chinese medicine pills to maximize formability and improve drying efficiency (e.g., to address issues such as drying cracks and crusting). It is evident that the drying process affects the internal structure, particularly the looseness and hardness, which are key factors determining the quality of the finished concentrated pills. The textural properties of the dried pills, such as the number and size of pores and capillaries, are closely related to water migration during dissolution [9], and it is worthwhile to investigate whether changes in drying conditions affect the active ingredients of the pills.

Hot air drying, vacuum drying, microwave drying, and combination drying are now employed for drying concentrated Chinese medicine pills. In the pharmaceutical sector, compartmentalized hot air drying technology is widely used, utilizing temperature differentials to transfer heat to materials [10]. Microwave drying employs a high-frequency electromagnetic field to generate heat through polar molecule vibration, vaporizing internal moisture for high drying efficiency [11]. A representative example of a widely used Chinese medicine in concentrated pill form is the LWDH pill, containing six herbs such as Prepared Rehmannia Root, which nourishes the liver and kidneys and contains volatile constituents like Danshi Phenol. The drying process is crucial for the dissolution timeframe and preservation of volatile components. Utilizing contemporary technology, this study examines how various drying techniques influence the texture and water migration in the LWDH pill, with the aim of improving dissolution performance, ensuring consistent quality, and enhancing clinical potential.

## 2. Materials and Methods

### 2.1. Materials

Hot air and microwave-dried Liuwei Dihuang (HAMD-LWDH) concentrated pills, hot air-dried Liuwei Dihuang (HAD-LWDH) concentrated pills, LWDH Raw Herbal Powder, and LWDH Extract were provided by Lanzhou Foci Pharmaceutical Co. (Lanzhou, China). All raw materials, excipients, and manufacturing processes were identical between the two types of pills, with the drying method being the only difference. Gallic acid, Morroniside, Loganin, Sweroside, and Paeonol were supplied by the China Institute of Food and Drug Control (CIFDC, Beijing, China), while Benzoylpaeoniflorin was sourced from Shanghai Yuanye Biological Co. (Beijing, China).

The following equipment and instruments were used: a DHG-9243BS-III Electric Blast Drying Oven (Shanghai CIMO Medical Instrument Co., Ltd., Shanghai, China), a YUJ-16C Microwave Vacuum Dryer (Huayuan Shangyu Technology Co., Ltd., Tianshui, China), a AccuPyc II 1340 Gas Pycnometer (Micromeritics, Norcross, GA, USA), a Mastersizer 3000 Laser Diffraction Particle Size Analyzer (Malvern Panalytical, Malvern, UK), a TA-HD plus 6049 Texture Analyzer (Stable Micro Systems, Godalming, Surrey, UK), a NMI20-025V-I Nuclear Magnetic Resonance Imaging and Analysis System (Niumag Co., Ltd., Suzhou, China), a Ci-L-FL Upright Fluorescence Microscope (Nikon, Tokyo, Japan), a FEI Quanta 250 Scanning Electron Microscope (FEI, Hillsboro, OR, USA), a SkyScan 1276 Micro-Computed Tomography (Bruker, Kontich, Belgium), and an Agilent 1260 High-Performance Liquid Chromatography system (Agilent Technologies Inc., Santa Clara, CA, USA).

### 2.2. Determination of Basic Physical Properties

Water content was determined by the drying method, the hardness of the concentrated pills was measured using a hardness tester, and the true density of the pills was determined with a true density tester. The volume of the pills was measured by the burette drainage method. To minimize error, 20 pills were tested, and porosity was calculated using Formula (1). The disintegration time limit was also determined. The basic physical properties of the concentrated pills were thus established.

(1) $$\varepsilon =\frac{\rho_{true}-\frac{m}{V_{1}-V_{2}}}{\rho_{true}}.$$

In the formula, *m* is the mass of the pills, *V*_1_ is the liquid level reading in the burette before the pills are added, *V*_2_ is the reading after the pills are added, *ρ_true_* is the true density of the pill, and *ε* represents porosity.

### 2.3. Study on the Effect of Material Properties on the Dissolution Time Limit of Concentrated Pills

Unlike traditional pills, the viscosity, moisture content, solubility, composition, and particle size distribution of the extract and raw herb powder, which are the main components of concentrated pills, significantly influence the pills’ textural properties and water migration during dissolution and dispersion. As a result, variations in the composition of the materials in concentrated pills may lead to changes in their texture during dissolution, affecting the dissolution time. To evaluate whether the changes occurring during the dissolution process of the extract and raw herb powder in LWDH pills have a dual effect, either facilitating or impeding the dissolution process, the following measurements were made: particle size of the raw herb powders after soaking, and the water solubility index of the infusions.

#### 2.3.1. Determination of Soaked Particle Size of Raw Herb Powders

Although the impact of raw herb powders on water migration and pill texture is complex, previous studies generally indicate that raw herb powders rich in plant fibers, starch, and other nutrients expand as they absorb water, thus aiding in the dissolution process. LWDH pills contain fine powder of yam and common macrocarpium fruit. To investigate whether the raw herb powder functions similarly to a tablet disintegrant during the dissolution of concentrated pills, the particle size of raw herb powder soaked for different durations was measured using a laser scattering particle sizing instrument. Changes in particle size were used to reflect the powder’s water absorption and expansion. To prevent the outflow of raw drug powder in the water due to the concentration difference between the inside and outside of the cells, a saturated solution of raw drug powder was used as the dispersing medium (100 mL per sample). The particle size of the raw drug powder immersed in water was measured at 0, 15, 30, 45, 60, 75, 90, 105, and 120 min, respectively (*n* = 3). The parameters of the laser scattering particle size instrument are as follows: sampling volume 3 mL (1 g of raw herb powder soaked in 100 mL of deionized water), dispersing medium 100 mL, mixing speed 2700 rpm, refractive index 1.65, absorption rate 0.001, background and sample scan time 10 s, and analysis mode generic.

#### 2.3.2. Determination of Water Solubility Index

To determine whether the dissolution of the extract plays a decisive role in the dissolution process of concentrated pills, the LWDH extract was crushed after drying under reduced pressure for 7 d. A sample of 1.5 g of extract powder, or ten pills of each type of LWDH prepared using the two drying methods, was accurately weighed and recorded as m_1_ (g). The sample was diluted with deionized water to 20 g, sealed with parafilm, magnetically stirred at 37 °C for 30 min, and then centrifuged at 4000 rpm for 10 min. The supernatant was transferred to an evaporating dish, evaporated using a water bath, and dried in an oven at 105 °C until a constant weight was reached. The residual solid mass in the sample was recorded as m_2_ (g) [12]. The water solubility index of the extracted powder was calculated according to Formula (2) (*n* = 3):

(2) $$Water\;Solubility\;Index=\frac{m_{2}}{m_{1}\times (1-Weight\;loss\;by\;powder\;drying)}\times 100\%.$$

### 2.4. Texture Analysis

The texture analyzer quantitatively measures the texture characteristics of the LWDH pills by monitoring the force applied to samples in real-time via force sensors, thereby objectively reflecting changes in the internal structure before and after dissolution [13,14]. LWDH samples, prepared using different drying methods, were randomly selected and placed in the sample cup. Based on Tomas J’s methodology that has been refined [15], the P5 probe was employed to press down on the pellet, and this was maintained for 100 s in a dry relaxation state. The degree of force reduction during this phase, expressed as ΔF_1_, provides an indication of the sample’s hardness. The greater the hardness, the more readily the probe reaches the set force, and the smaller the observed decrease in detected force. To assess internal force changes during pill disintegration, 20 mL of deionized water was rapidly added to the sample cup after 100 s. A rapid change in the force detected by the probe was observed 10 s after the water was introduced. The degree of force reduction at this stage was recorded as ΔF_2_, indicating that the dry pills were quickly dissolved by the surface paste upon contact with water molecules. Subsequently, a gradual change in force occurred, expressed as ΔF_3_, signifying the dissolution of the pill’s outer layer by the absorbed water molecules. The texture analysis was performed in stress relaxation mode with a pre-test speed of 6.0 mm/min, a mid-test speed of 9.0 mm/min, and a post-test speed of 600.0 mm/min, with a force of 700.0 g and a time of 10.0000 min. Despite deviating from previously reported values, this combination produced the smallest RSD values for force differences through orthogonal experimentation, all of which were below 10%. This indicates that 700 g is the most suitable force level for this test system.

### 2.5. Dissolution and Dispersion Kinetics of LWDH Pills

Samples of 0.17 g HAWD- and HAD-dried LWDH pills were placed into vials containing 0.9 g of deionized water. Digital images were captured at 0, 15, 30, 45, 60, 75, 90, 105 and 120 min during the dissolution process, creating a visual record of pill dissolution over time. Additionally, Low-Field Nuclear Magnetic Resonance (LF-NMR) was used to quantitatively analyze the dissolution kinetics of the concentrated pills [16]. The LWDH samples were placed in 25 mm diameter NMR sample tubes, each containing 0.9 g of deionized water. A T_2_ map was generated after signal inversion, following the acquisition of an empty tube signal using the CPMG sequence, and then the sequential acquisition of the sample signal at different time points. Moreover, low-field MRI imaging was utilized to dynamically observe the water penetration into the LWDH pills at various stages [17,18]. T_1_-weighted images of the samples were sequentially obtained at different time points using MRI imaging software (NMImaging-V1.23.02D) to produce imaging maps of the pills throughout the dissolution process. Horizontal and vertical lengths were measured using the built-in caliper tool within the MRI imaging software, with the outermost edges of the pellet used as standardized reference points. All measurements were performed in triplicate to obtain the average value. The main parameters of the CPMG sequence are as follows: sampling frequency 200 kHz, hard pulse 90° with a pulse width of 7.00 μs, hard pulse 180° with a pulse width of 12.48 μs, repeat sample waiting time 5000 ms, number of scans two, echo time 0.4 ms, and number of echoes 18,000. MRI imaging parameters: layer 1, layer thickness 9 mm, number of acquisitions six, field of view 45 × 45 mm, TR 800 ms, TE 5.9 ms, frequency direction 256, and number of encoding steps 192.

### 2.6. Study on the Microstructure of LWDH Pills

#### 2.6.1. Microstructural Observations

It is widely accepted that during the dissolution and disintegration process, water infiltrates concentrated pills through the pores, disrupting the skeletal structure with the assistance of capillaries [19]. Therefore, the dissolution time of concentrated pills is related to the number and size of pores and capillaries present within the pills. The cavities and pores in porous solids that are open to the external environment are termed open pores. These include cross-linked pores, through pores, and blind pores. The surface area of these pores can be analyzed through gas adsorption, which facilitates moisture entry [20]. Additionally, there may be closed pores within the solid that are not connected to the outer surface and are inaccessible to fluids. Such closed pores are not detectable by gas adsorption or mercury intrusion techniques; moisture must first dissolve the surface paste before reaching these pores. The microstructure of the LWDH pill cross-section was assessed based on the looseness of the internal structure and the difficulty of water entry [21,22]. Rounded LWDH samples were selected for paraffin embedding and subsequently sliced into 300 sections with a thickness of 3 μm per slice to obtain the pellet cross-section. The pellet cross-section was examined under a microscope at 4× magnification. Following paraffin sectioning, the pellets were coated with gold, and the morphology of the pill cross-section was analyzed using scanning electron microscopy.

#### 2.6.2. Microstructural Analysis

Micro-CT imaging can reveal the overall pore structure of LWDH pills [23]. Randomly selected LWDH samples underwent micro-CT structural scanning to compare the pore distribution, open pore volume, and closed pore volume of LWDH pills prepared using various drying processes. Micro-CT scanning parameters were: ray tube current 200 μA, voltage 70 kV, scanning of the whole object, scanning resolution 5.033940 μm, exposure time 350 ms, scanning angle 180°, and scanning of the body model (phantom; provided in a uniform configuration by the equipment manufacturer) under the same conditions for calibration.

### 2.7. Chemical Composition Analysis Based on HPLC Fingerprints

HPLC was employed to identify the chemical fingerprints of LWDH pills produced using different drying methods, allowing for the comparison of the effects of these procedures on the contents of Gallic acid, Morningside, Loganin, Sweroside, Paeonol, and Benzoylpaeoniflorin. The HPLC method was as follows: the column was a Diamonsil-C18 reversed-phase column; the column temperature was 30 °C; the volumetric flow rate was 1 mL/min; the injection volume was 10 μL; the mobile phase was A (acetonitrile)—D (0.1% phosphoric acid aqueous solution). Gradient elution: 0–15 min, 2~15%A; 15—25 min, 15~25% A; 25—40 min, 25~70% A; 40—60 min, 70~95% A. Detection wavelengths were 235 nm for fingerprinting and 270 nm for Paeonol.

## 3. Results

### 3.1. Results of Basic Physical Properties

**Figure 2:**
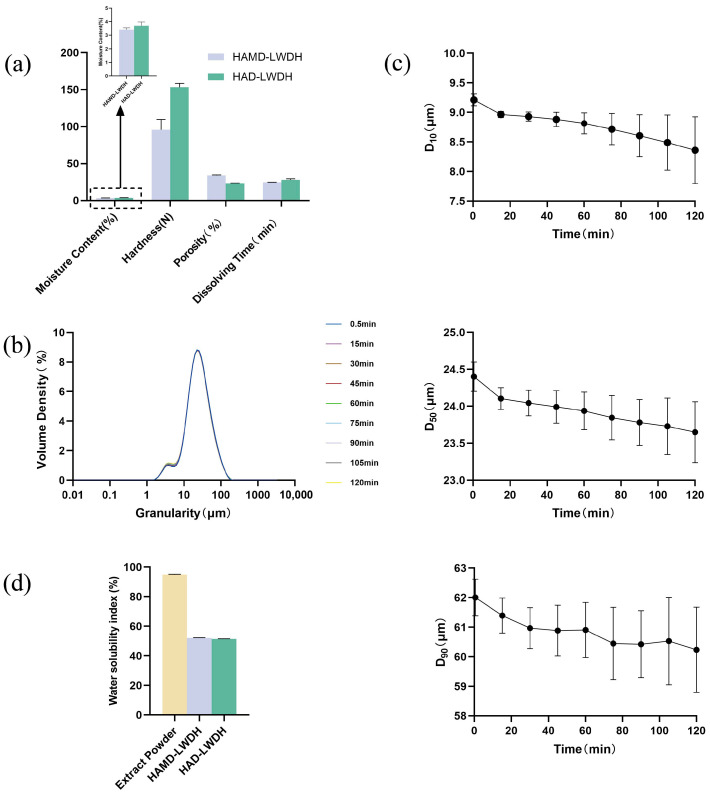
Diagrams of the physical properties of LWDH pills. (**a**) Basic physical property diagram; (**b**) powder particle size distribution; (**c**) crude drug powder soaked over different times with effects on particle size change; (**d**) water solubility index diagram.

Figure 2 shows the physical properties of LWDH pills. The water content of the pills produced using the two different drying methods was roughly equivalent, as illustrated in Figure 2a. However, HAMD-LWDH pills exhibited lower hardness, disintegrated more rapidly, and possessed greater porosity compared to HAD-LWDH pills. The observed difference in hardness between HAWD-LWDH and HAD-LWDH pills is primarily attributed to the drying method, but it is not solely the drying process per se; it arises from the microstructural modifications induced by the drying conditions. Specifically, microwave-assisted drying (HAWD) promotes rapid water removal, leading to the formation of a more porous internal network and smaller, more interconnected cavities. In contrast, conventional hot-air drying (HAD) results in slower moisture removal, producing a denser matrix with fewer internal voids.

### 3.2. Influence of Material Properties on the Dissolution Time of LWDH Pills

LWDH powder is produced by grinding Chinese yam and a portion of Common Macrocarpium Fruit. The fine powder derived from yam is rich in starch, comprising 45% to 64% of the total biomass of Chinese yam (in dry mass) [24]. Additionally, yam contains a variety of biologically active compounds, such as mucopolysaccharides and polyphenols. Yam starch swells at elevated temperatures; however, the drying temperature during preparation is precisely maintained within the range of 72 °C to 84 °C, which corresponds to the pasting temperature [25]. During this process, the protein components in yam powder bind to starch granules via hydrogen bonds, electrostatic forces, and van der Waals forces, or they disperse in the gaps between starch granules [26]. This specific temperature range results in tight internal bonding, thereby affecting the dissolution properties [27,28]. Common Macrocarpium Fruit contains polysaccharides (6.6% to 25.2%), with concentrations increasing after processing into yellow wine. The molecular structure of these polysaccharides facilitates moisture absorption and water redistribution within the pills due to the presence of hydroxyl, carbonyl, and other functional groups that form hydrogen bonds with water [29,30]. When LWDH powder is immersed in a saturated solution, the particle size decreases with increasing immersion time, although the reduction is not readily observable (Figure 2b,c). This phenomenon may be attributed to the minimal difference in osmotic pressure inside and outside the plant cells, where the limitations of the cell wall prevent water from substantially increasing the size of the plant cell. During the stirring process, pressure from the saturated solution may extrude the cells, leading to a reduction in particle size. This suggests that the water-absorbing expansion of the powder does not significantly influence the dissolution of concentrated pills.

LWDH extract is obtained through the water extraction, concentration, and drying of certain tablets (including Peony bark, Prepared Rehmannia root, Common Macrocarpium Fruit 20 g, Poria cocos, and Rhizoma alismatis), which primarily consist of water-soluble components. As illustrated in Figure 2d, the water solubility index of LWDH extract powder exceeds 84%, indicating that the extract powder is highly soluble in water. The dissolution of the LWDH extract plays a critical role in the overall dissolution process of the concentrated pills.

### 3.3. Results of Texture Analysis

**Figure 3:**
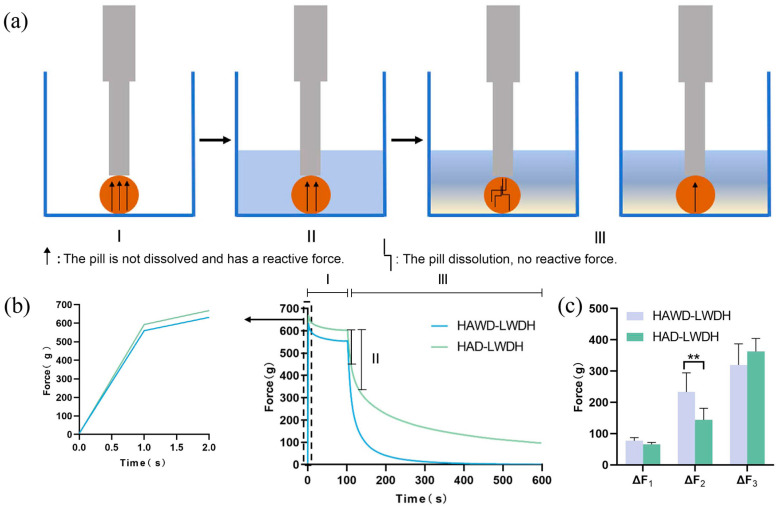
LWDH pill texture diagrams. (**a**) Schematic diagram of the textural process: I is the dry relaxation stage, II is the stage of rapid decrease in force after addition of water, III is the slow wet relaxation stage of HAWD-LWDH pills, and the slow wet relaxation stage of HAD-LWDH pills; (**b**) texture curve: I is the dry relaxation stage, II is the stage of rapid decrease in force after the addition of water, and III is the stage of slow wet relaxation; (**c**) the bar graph of ΔF_1_, ΔF_2_, and ΔF_3_, ** *p* < 0.01.

To analyze the change in its internal force throughout the dissolution of the pill, 20 mL of deionized water was added to the sample cup after 100 s of dry relaxation, and the wet relaxation of the pill was recorded for the entire duration of 10 min (Figure 3a). The resulting curve illustrates the force required to maintain the probe’s displacement at each time point during maximum force application (Figure 3b and Table 1). The force values for each stage of the texture analysis process are shown in Figure 3c. To some extent, ΔF_1_ represents the hardness of the pills. A larger ΔF_1_ indicates a greater degree of downward pressure exerted by the probe, reflecting reduced hardness and a looser internal structure of the pills. Consequently, the internal hardness of HAWD-LWDH pills is lower, as indicated by a smaller ΔF_1_, and its downward displacement (0.036 mm) exceeds that of HAD-LWDH (0.032 mm) pills. These structural differences at the microscopic level directly affect the mechanical response of the pills under probe pressure. During water dissolution, the internal pores of the pills collapse, leading to the progressive destruction of the skeletal structure and a gradual dissolution process. Higher ΔF_2_ and ΔF_3_ values indicate more extensive water penetration and dissolution. The ΔF_2_ of HAWD-LWDH pills is greater than that of HAD-LWDH pills, likely due to their increased porosity facilitating faster early-stage water infiltration and dissolution of the surface layer. Conversely, the ΔF_3_ of HAWD-LWDH pills is smaller than that of HAD-LWDH pills, reflecting more pronounced initial internal collapse, after which the force rapidly decreases to 0 g, indicating complete separation of the skeletal structure from the probe. Overall, these data suggest that the microwave drying process enhances pill porosity, reduces internal hardness, and accelerates dissolution, highlighting a clear mechanistic link between drying-induced microstructure and functional performance.

### 3.4. Study on the Dissolution Kinetics of LWDH Pills

#### 3.4.1. LF-NMR T_2_ Relaxation Time During Dissolution and Dispersion of LWDH Pills

**Figure 4:**
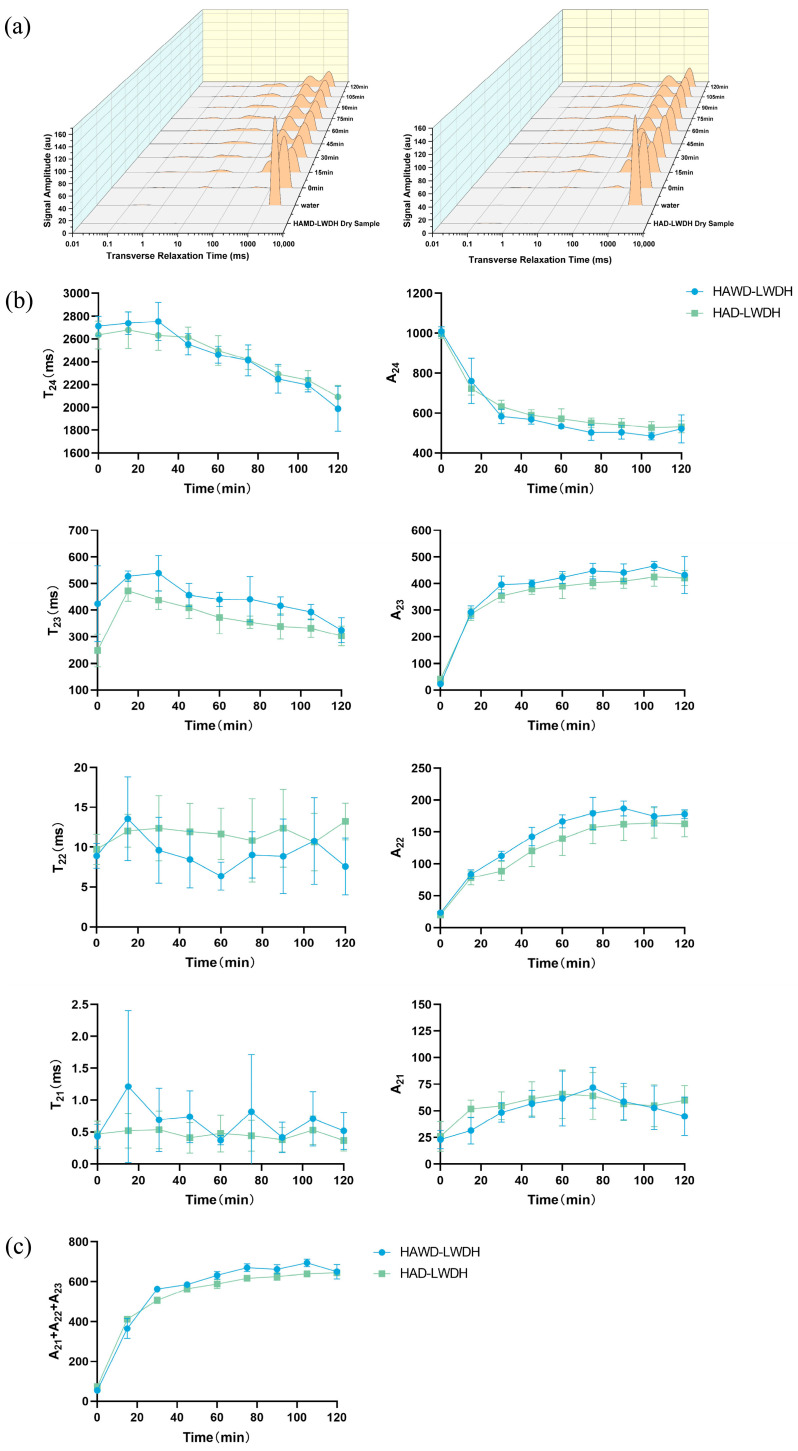
LWDH LF-NMR T_2_ spectrum. (**a**) Transverse relaxation diagram; (**b**) water relaxation and peak area diagram of each phase; (**c**) water absorption kinetics diagram.

Figure 4 shows the change in T_2_ transverse relaxation of LWDH pills during dissolution. The T_2_ transverse relaxation time in LF-NMR refers to the duration it takes for the magnetic resonance signal to diminish in the magnetic field; a shorter T_2_ time indicates a more rapid decay of the magnetic resonance signal. The four peaks that comprise the T_2_ relaxation pattern symbolize the water that penetrates the interior of the pills at various time intervals. The peak of bound water, T_21_, which ranges from 0.01 to 1 ms, represents water that is tightly associated with the pill’s ingredients. T_22_, ranging from 1 to 10 ms, denotes water that has limited mobility. The peak between 1 and 10 ms represents water that is adsorbed onto the inner layer of particles following the dissolution of the pill. The peak of free water, T_23_, ranging from 10 to 100 ms, corresponds to water present in the internal capillaries of the pill and in the cellular interstitial space of the raw herb powder. T_24_, which spans from 100 to 10,000 ms, represents water that is not bound to the various components of the pill. A longer transverse relaxation time indicates improved mobility of the water in this phase, and a larger peak area corresponds to higher water content in that phase [31]. As illustrated in Figure 4a, with increasing dissolution time, water progressively infiltrates the pill and interacts with its various components. Consequently, the unbound water content gradually decreases, while the content of bound water, semi-bound water, and free water increases, ultimately reaching equilibrium by the end of the dissolution period.

Both the T_24_ and A_24_ of LWDH pills progressively decreased with increasing dissolution time, indicating a reduction in the mobility and content of unbound water, which became more closely bonded to the pill, as shown in Figure 4b [32]. During the initial dissolution phase (0–30 min), free water rapidly diminished while the pill quickly absorbed water. Absorption occurred swiftly. Water absorption by HAWD-LWDH pills exceeded that of HAD-LWDH pills. Between 30 and 120 min, unbound water content gradually decreased until a stable state was achieved. Throughout this period, water absorption of HAWD-LWDH pills consistently surpassed that of HAD-LWDH pills, demonstrating that HAWD-LWDH pills facilitated easier water absorption during dissolution, allowing water to penetrate the pill via capillary action, thereby breaking down components and reducing unbound water content.

The T_23_ of LWDH pills exhibited an initial increase followed by a decrease under various drying conditions, while A_23_ continued to rise. This pattern suggests that the free water content of the tablets increased, and their fluidity improved during the early stages of disintegration and dispersion. Over time, free water dissolved the components and infiltrated deeper into the pills via capillary tubes, eventually achieving equilibrium between internal and external moisture levels. During the 0–30 min interval, the T_23_ and A_23_ of the pellets from both drying methods increased rapidly, with the T_23_ and A_23_ of HAWD-LWDH pills being higher than those of HAD-LWDH pills. This indicates that water entered HAWD-LWDH pills more quickly and in greater volumes [33].

During the dissolution process, the T_22_ of LWDH pills exhibited significant fluctuations between the two different drying methods; however, A_22_ displayed an upward trend for both methods. This indicates that water penetrated the interior of the pill through capillary action, resulting in a gradual increase in the content of non-flowing water [34]. Notably, HAWD-LWDH pills demonstrated a higher A_22_ compared to HAD-LWDH pills, suggesting that the texture of HAWD pills is more conducive to water absorption into the pill body. The presence of larger voids within HAWD pills facilitates the generation of more non-flowing water, characterized by a lower concentration and smaller T_22_, thereby enhancing fluidity.

The T_21_ value of LWDH pills remained relatively stable, while A_21_ initially increased before decreasing. Both T_21_ and A_21_ rose rapidly from 0 to 75 min, indicating that water gradually entered the pill and formed close associations with its components during this period. Subsequently, A_21_ experienced a slight decrease, likely attributed to the differing rates at which external water infiltrated the pill compared to the rate of dissolution of the components. Eventually, the internal water of the pill became fully saturated, resulting in the stabilization of both T_21_ and A_21_.

#### 3.4.2. Absorption Kinetics of the Dissolution and Dispersion Process of LWDH Pills

In the LF-NMR T_2_ pattern, A_21_, A_22_, and A_23_ represent the water content in distinct phases throughout the dissolution process of the pill. Therefore, A_21_ + A_22_ + A_23_ is utilized to denote the quantity of water absorbed at different LWDH pill dissolution times, referred to as A_2_. Figure 4c illustrates the temporal changes in water absorption during the dissolution of LWDH pills; A_2_ increased rapidly during the initial 30 min, followed by a gradual rise that approached equilibrium after 90 min, with A_2_ for HAWD-LWDH pills exceeding that of HAD-LWDH pills. The paired-sample “*t*”-test findings revealed that the A_2_ values between HAWD-LWDH and HAD-LWDH pills showed a statistically significant difference during the 30–120 min period (*p* = 0.006 < 0.05), showing that HAWD-LWDH pills had a better capacity for water absorption. However, no significant difference was observed between the two samples during the initial 0–30 min period (*p* = 0.927 > 0.05), which can be attributed to the fact that water must first wet the surface of the pellets during the early stage of dissolution, during which both samples absorbed significant amounts of water.

The water absorption data during the dissolution of LWDH pills were fitted using the Weibull model, Peleg model, and double exponential model, with the results presented in Table 2. The Weibull model emerged as the best-fitted model, as indicated by a R^2^ value approaching one, a residual sum of squares (RSS) close to zero, and a lower Akaike Information Criterion (AIC) value, all of which suggest a superior model fit. In the Weibull model, the parameter “a” represents the maximum cumulative release, reflecting the theoretical equilibrium dissolution amount and the water-holding capacity of the pill’s pore structure. A larger “a” value indicates greater water absorption capacity. HAWD-LWDH pills exhibited a larger *a* value than HAD-LWDH pills, suggesting a higher capacity for water accommodation. The parameter *c* is a time scale parameter negatively correlated with the diffusion rate of pellet components; a larger “c” value corresponds to faster dissolution. HAWD-LWDH pills demonstrated a larger “c” value than HAD-LWDH pills, indicating a faster component diffusion rate during dissolution. These differences highlight the influence of drying methods on the dissolution behavior of the pills (Table 3).

#### 3.4.3. Visualization and LF-NMR Imaging of the Static Dissolution Process of LWDH Pills

**Figure 5:**
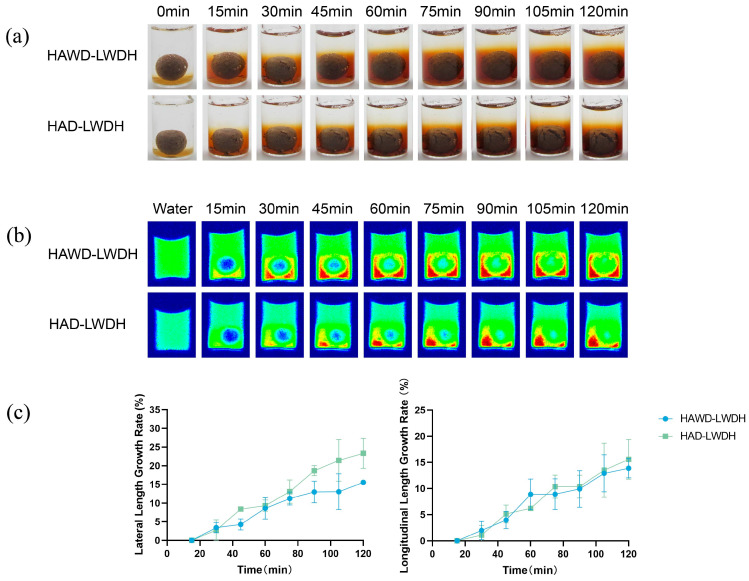
Dynamic dissolution process of LWDH pills with different drying methods. (**a**) MRI imaging; (**b**) visuals; (**c**) lateral length growth rate and longitudinal length growth rate.

Figure 5 shows the dynamic dissolution process of LWDH pills with different drying methods. The volume of LWDH pills increased gradually with the dissolution and dispersion processes. Cracking was observed on the surface, and the diffusion layer formed by the dissolution of the pill components in water became progressively thicker. Notably, the diffusion layer of HAWD-LWDH pills was consistently thicker than that of HAD-LWDH pills at all time points in Figure 5a. The surface of HAD-LWDH pills exhibited fractures, and water penetration occurred slowly, resulting in a slower dissolution rate, sparse texture, and an overall increase in volume.

The blue-to-red color gradient in the MRI imaging suggests that moisture binds increasingly tightly to the pill components, while the lateral relaxation time of pure water is longer, resulting in a blue-green hue [35]. As shown in Figure 5b, with the extension of the dissolution time, water gradually entered the pill to interact with its ingredients. Concurrently, the cells of the extract or raw herb powder diffused into the surrounding water along the concentration gradient, leading to a reduction in the transverse relaxation time of this portion of the water. This was initially manifested as the appearance of a yellow diffusion layer in the imaging map. Yellow diffusion layers were observed in both HAWD-LWDH and HAD-LWDH pills at 15 min, indicating that some ingredients had dissolved in the water. As additional components of the pill gradually dissolved, a red diffusion layer emerged. Eventually, water completely entered the HAWD-LWDH pill by 90 min and the HAD-LWDH pill by 105 min. At this point, the blue portion of the pill disappeared, and the overall hue shifted to green. The concentration of dissolved components in the lower water layer, influenced by gravity, resulted in a thicker red diffusion layer, whereas the upper water layer, which contained fewer dissolved components, appeared green. Additionally, because HAWD-LWDH pills have a larger porosity than HAD pills and their ingredients diffuse in water more quickly, water enters the HAWD-LWDH pill and fills the pill’s pore space. In contrast, HAD-LWDH pills have a compact texture, and water enters HAD-LWDH pills and then moves to the center of the pill through capillary action and pore space expansion [36], resulting in a phenomenon where the transverse and longitudinal lengths of HAD-LWDH pills grow at a faster rate; refer to Figure 5c.

### 3.5. Microstructure Observation

**Figure 6:**
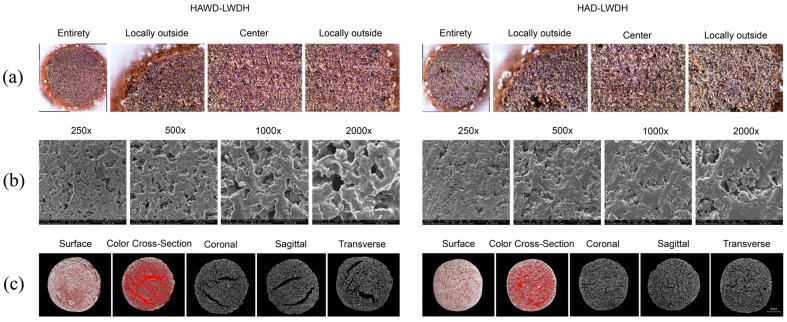
LWDH microstructure diagrams. (**a**) Transverse section microstructure diagrams; (**b**) cross-section scanning electron microscope diagrams; (**c**) micro-CT images—the red parts represent pores.

Figure 6 shows the microstructure of LWDH pills. Figure 6a illustrates that the internal colors of LWDH, produced through different drying methods, varied yet exhibited a more uniform appearance. HAWD pills displayed a reddish-brown color, while HAD pills appeared yellowish-brown. This variation may be attributed to the influence of the drying method on the chemical composition of the pills, consequently affecting the cross-sectional color [37]. Additionally, differences in the drying methods resulted in distinct internal textures; HAWD-LWDH pills contained more numerous and larger pores compared to HAD-LWDH pills, as shown in Figure 6b.

Micro-CT results indicated that the internal pores of the pellets are represented by the red areas in Figure 6c. Open pores were identified and quantified using micro-CT image analysis. After three-dimensional reconstruction of the scanned samples (NRecon V 1.7.4.2), a pore network analysis was performed using image processing software (CT Analyser 1.20.3.0). Open pores were defined as pores that are connected to the external surface of the sample, allowing fluid penetration. To discern open pores, the external surface of the sample was first segmented, and a “connected component labeling” algorithm was applied to identify all pore voxels that were connected to the exterior. Pores that did not connect to the external surface were classified as closed pores. The open porosity was then quantified as the volume fraction of open pores relative to the total sample volume. HAWD-LWDH pills exhibited large internal pores with volumes of up to 6.70, 6.60, and 5.88 mm^3^, an average pore size of 0.06 mm, an opened pore volume of 25.44 mm^3^, and a total porosity of 15.67% (Table 4). These findings suggest that the HAWD pellets were more susceptible to moisture intrusion, as indicated by both total porosity and open pore volume. Pore connection density, defined as the number of pore connections per unit volume or surface area, is indicative of fluid propagation performance within the medium. Pore connection density was measured based on the pore network model extracted from the micro-CT data. Following three-dimensional reconstruction and pore segmentation, the pore space was skeletonized to generate a network of nodes (pore bodies) and links (pore throats). The connection density was then calculated as the number of connections (or pore throats) per unit volume of the sample. A high pore connection density reflects tighter pore connections and enhanced fluid propagation, whereas a low pore connection density indicates sparser pore connections and inferior fluid propagation capabilities [38]. Table 4 demonstrates that the pore connection density of HAWD-LWDH pills is higher than that of HAD-LWDH pills. This suggests that the internal structure of the pellets is more significantly influenced by microwave drying during the second stage of the hybrid drying method, thereby facilitating dissolution [39].

### 3.6. HPLC Fingerprint Analysis

**Figure 7:**
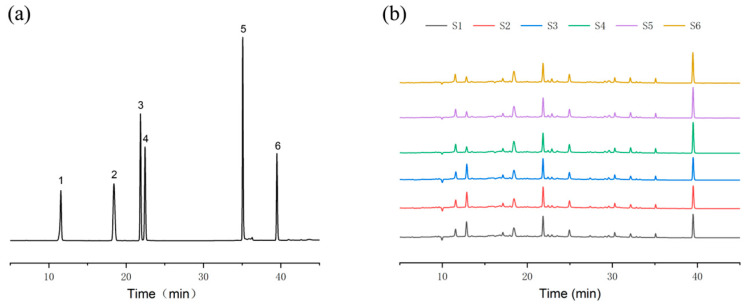
**1:** Gallic acid; **2:** Morroniside; **3:** Loganin; **4:** Sweroside; **5:** Benzoylpaeoniflorin; **6:** Paeonol. (**a**) Mixed reference substance spectrum; (**b**) fingerprint spectrum.

Figure 7 shows the mixed reference substances and fingerprints of LWDH pills. The two LWDH samples were fingerprinted under the chromatographic conditions of “2.7”, resulting in liquid phase patterns for six batches of samples. These liquid phase patterns were analyzed using the “Traditional Chinese Medicine Chromatographic Fingerprint Similarity Evaluation System” (2012 version), generating fingerprint patterns. According to the established standards, peak one was identified as Gallic acid, peak two as Morroniside, peak three as Loganin, peak four as Sweroside, peak five as Benzoylpaeoniflorin, and peak six as Paeonol (Figure 7). Similarity was calculated following peak matching using the “median” method, yielding similarities of 0.995, 0.995, 0.995, 0.995, 0.997, 0.998, and 0.998 for samples S1 to S6, respectively (S1, S2, S3: HAWD-LWDH pills; S4, S5, S6: HAD-LWDH pills). These results indicated that the fingerprint profiles of the six batches of LWDH samples were highly similar, with no significant differences in composition among the samples.

The Chinese Pharmacopeia designates Paeonol as an indicator component for the LWDH content test, specifying a minimum content of 1.78 mg/g. The volatile component Paeonol was measured at 2.2570 mg/g for HAWD-LWDH pills and 3.1601 mg/g for HAD-LWDH pills. Although the Paeonol content in the pills produced by both drying methods met the 2020 Chinese Pharmacopeia requirements, it is evident that the HAD pills contained a higher salvinorin content than the HAWD pills. This suggests that different drying methods affect the chemical composition of the pills.

## 4. Discussion

This study establishes a multidimensional characterization system of “texture properties and water migration” for concentrated pills, using LWDH as a model drug. The research encompasses the morphological transition from powder to pill, macroscopic to microscopic structural analysis, and the static to dynamic monitoring of the dissolution and dispersal processes. This provides robust technical support and extensive experimental data to elucidate the mechanisms of dissolution and dispersal.

Testing has demonstrated that the primary dissolution mechanism of LWDH involves the dissolution and diffusion of the paste on the surface of the pills. The pills gradually dissolve, and the powder particles disperse in water, completing the dissolution and dispersal process from the outside in, similar to the etching and dissolution process of Chinese medicine half-extract tablets. However, the high viscosity of the dissolved extract may hinder water from penetrating the pill. Furthermore, the presence of raw herb powder in concentrated tablets has a complex effect on pill texture and water migration. Contrary to previous studies, which generally posit that raw herb powder facilitates disintegration, the findings of this study indicate that its addition results in the formation of solid body textural characteristics within the pill, leading to a reduction in pore space that impedes water from easily entering the pill body. Different drying methods influence the degree of looseness within the internal structure of the tablets, thereby affecting water migration during the dissolution process. Consequently, the texture and dissolution characteristics of the pills during drying, along with the dynamics of water migration, play critical roles in determining the dissolution capability and regulatory processes of the pellets.

The majority of concentrated pills exhibit a slow dissolution time due to several factors, including the nature of the raw powder, particle size, excipients, method of preparation, drying process, and water content of the final pills. Adjusting the drying process has a minimal effect on controlling the dissolution time of the pills relative to these factors. Four general stages can be distinguished in the drying process: preheating, constant speed, decreasing speed, and balancing. Hot air drying effectively fixes the volume of the pills and removes the majority of the water during the constant speed stage. The rate at which the remaining water migrates to the surface is slower than the rate at which surface water evaporates, indicating that the pills are approaching the deceleration stage. Subsequently, hot air drying and evaporation become less effective. Microwave drying, on the other hand, utilizes polarized water molecules to alter their position within an electromagnetic field, generating friction that releases heat. This method effectively and rapidly removes water from samples that have been heated to high temperatures, promoting the formation of a loose, porous structure in the material during the drying process, which aids in the dissolution of the pills.

Chinese medicine pills possess multi-component characteristics, with most containing heat-sensitive components that are prone to decomposition at elevated temperatures. The traditional static hot air drying process is time-consuming and can lead to the loss of heat-sensitive components, negatively affecting production efficiency [40]. In contrast, the dynamic microwave drying process heats from the inside out, offering high drying speed and efficiency. However, this rapid migration of a significant volume of water can cause a substantial expansion of the pills, resulting in cracking and color discrepancies, which severely impacts the quality of the final product. Experimental results indicated that a combination of hot air and microwave drying methods was beneficial. Initially, hot air drying reduces moisture while fixing the form and size of the pills. This is followed by microwave drying, which facilitates the formation of continuous pore channels from the inside out, thereby increasing porosity and promoting the migration of water during the dissolution process. Importantly, this combined drying approach did not lead to significant changes in the chemical composition of LWDH pills. Consequently, the utilization of hot air and microwave drying was employed to further regulate the drying process parameters, serving as an effective control method to enhance the dissolution time and quality of concentrated pills.

## Figures and Tables

**Table 1:** Fitting of texture equation of LWDH pills with different drying methods (*n* = 3).

Samples	Function	Mathematical Equation	R^2^
HAWD-LWDH	Logarithm	y1 = −17.22ln(x) + 630.4	0.9959
	Linear	y2= −27.774x + 588.69	0.9909
	Exponent	y3 = 104.68e^−0.01x^	0.9592
HAD-LWDH	Logarithm	y1= −14.72ln(x) + 668.3	0.9974
	Linear	y2 = −16.704x + 621.25	0.9891
	Exponent	y3 = 296.04e^−0.003x^	0.9378

**Table 2:** Fitting results of A_2_ water absorption kinetics (*n* = 3).

Model	Parameter	HAWD-LWDH	HAD-LWDH
Peleg	R^2^	0.244	0.205
	RSS	260,262.24	212,432.70
	AIC	42.11	41.50
Biexponential	R^2^	0.986	1.000
	RSS	4757.55	127.61
	AIC	30.11	19.25
Weibull	R^2^	0.992	0.993
	RSS	2820.79	1999.68
	AIC	28.54	27.51

**Table 3:** Parameters of Weibull model fit for concentrated pills.

Parameters	HAWD-LWDH	HAD-LWDH
a	617.39	554.256
b	−2.207	7.314
c	0.251	0.106
d	0.199	0.508

a: maximum cumulative release; b: Initial burst release; c: time scale parameter; d: Shape parameter.

**Table 4:** Pore distribution table of LWDH pills with different drying methods.

Samples	Closed Pore Volume (mm^3^)	Opened Pore Volume (mm^3^)	Porosity (%)	Hole Connection Density (1/mm^3^)
HAWD-LWDH	2.1284	25.4357	15.6733	739.7949
HAD-LWDH	3.8545	15.1733	12.2548	314.6559

## Data Availability

The original contributions presented in this study are included in the article. Further inquiries can be directed to the corresponding author.

## Abbreviations

The following abbreviations are used in this manuscript:

LWDH	Liuwei Dihuang
LF-NMR	Low-Field Nuclear Magnetic Resonance
HPLC	High-Performance Liquid Chromatography
HAMD-LWDH	Hot air and microwave-dried Liuwei Dihuang
HAD-LWDH	Hot air-dried Liuwei Dihuang
RSS	Residual Sum of Squares
AIC	Akaike Information Criterion
